# New horizons in community engagement: Virtual community engagement studios amplifying community voices about health research in New Mexico

**DOI:** 10.1017/cts.2024.608

**Published:** 2024-10-02

**Authors:** Cynthia M. Killough, Julia Martinez, Holly Mata, Donna Sedillo, Pilar Sanjuan, Alexandra Roesch, Christopher Hudson, Brenda Bishop, Jose Gonzalez, Heidi Rishel Brakey, Nancy Pandhi

**Affiliations:** 1 University of New Mexico Health Sciences, Clinical and Translational Science Center, Albuquerque, NM, USA; 2 Otero County Community Health Council, Alamogordo, NM, USA; 3 McKinley Community Health Alliance, Gallup, NM, USA; 4 Quay County Health Council, Tucumcari, NM, USA; 5 Taos Health Council, Taos, NM, USA

**Keywords:** Community engagement, health research, clinical trials, underserved populations, underrepresented populations

## Abstract

There is increasing recognition of the crucial need for robust community engagement in health research and clinical trials. Despite this awareness, challenges persist in bridging the gap between researchers and communities. Much of the current discourse focuses on addressing issues such as cultural humility and equitable partnerships. To expand this conversation, we conducted community engagement studios, following the model by Joosten et al. We wanted to gather perspectives on research involvement across New Mexico. This process and resultant findings offer valuable insights into effective community engagement practices and advance clinical and translational science by amplifying community voices and needs.

## Introduction

In recent years, the imperative for robust community engagement in health research and clinical trials has become increasingly evident, with a growing recognition of its pivotal role in building trust, overcoming historical barriers to research participation, and advancing equity [1–4]. Despite this recognition, numerous challenges persist. Mistrust of research, historical trauma, cultural and linguistic barriers, policy and structural barriers are just some of the many challenges researchers still face as they strive toward effective community engagement [5–7].

Current discourse in the field of community engagement in research is particularly focused on addressing key issues such as cultural humility, equitable partnerships, and meaningful involvement of diverse communities [4,5,8,9]. In a recent study analyzing detailed trial records from all US clinical trial studies registered in ClincialTrials.gov in March of 2020, researchers found that people from minoritized populations were still underrepresented in trials, with a majority of studies not reporting race/ethnicity data [10]. At the University of New Mexico Health Sciences Center (UNM HSC), the Clinical and Translational Science Center (CTSC) supports a community engagement liaison specialist (CELS) position in order to address key issues and overcome barriers to research participation. The CELS is a community-based staff person who builds relationships and networks with community organizations, agencies, and community members on a regular basis in hopes of bridging the gap in diverse participation in research that plagues health research and clinical trials efforts. One avenue of continuous community engagement for the CELS is through consistent attendance at various New Mexico County and Tribal Health Councils.

Health councils, mandated by the state of New Mexico, collaborate with communities to assess overall health of community members, identify priorities, and work with partners to find solutions and resources [11]. In addition, councils advise local government on health policy. There are 33 active county community health councils and 9 tribal health councils in New Mexico. These councils offer networking and collaboration opportunities. The CELS currently attends ∼12 community health councils monthly statewide.

To identify individual and community needs for research participation in New Mexico, the CELS invited New Mexico community health council members to share their unique perspectives in a community conversation, also known as community engagement studios [12–14].

### Community engagement studios with New Mexico Health Councils

Developed by Joosten et al., community engagement studios (“CE studios”) bring community members to researchers for a structured consultative session about their research project [13,14]. The community members are well experienced and knowledgeable on the topic. This model brings community members “front and center,” refers to them as “experts,” and highlights their importance and potential impact to the project. We adapted this model to focus on research with communities generally, rather than one specific research project, but adhered to the model otherwise.

CE studios are preparatory to the project and therefore many insitutional review boards (IRBs) (including UNM’s) agree that studios do not need IRB approval as long as the studio is conducted with the following practices: audio recordings are allowed for note taking purposes only, may not be shared outside the studio facilitation team, and must be destroyed upon completion of notes; no transcription or data analysis may occur other than descriptive statistics of demographics and feedback survey results; and no direct quotes may be published.

The experts we asked to participate in our CE studios were health council members. The New Mexico Department of Health divides the state into four public health regions for different contexts: Northeast, Southeast, Southwest, and Northwest. Counties are further segmented within these regions. Leveraging this regional structure, the CELS identified health councils she actively attends in each region. She then invited individuals from those health councils to participate in virtual CE studios that would be hosted via the Zoom platform. The goal was to engage ∼8 participants per region per studio (8 participants × 4 public health regions = 32 experts).

As part of inviting experts to participate in a studio, all interested community health council members were asked to complete a brief demographic survey. Upon survey completion, the CELS confirmed participation interest and provided an individualized orientation to give the experts an overview of what to expect in the CE studio. During this orientation, the CELS also assessed accessibility accommodations, computer and internet access, and answered any questions related to using the virtual Zoom platform.

CE studios were conducted between March and June 2023. The CELS collaborated with qualitative methodologists at the UNM HSC CTSC Community Engagement and Research Core [15] to facilitate each studio conversation. The studios followed a uniform format across all sessions. This format consisted of: 1) a brief presentation by the CELS to outline the studio’s purpose; 2) structured questions asked by a facilitator; 3) an open discussion for experts to share additional perspectives; and 4) completion of an evaluation form by experts.

The CTSC Integrating Special Populations committee [16] helped the CELS write four primary questions to ask at each CE studio: 1) What does “research” mean to you? 2) What experience do you have with research? 3) What would communities want to know before agreeing to participate in research? and 4) When a study is presented as an opportunity for your community, what things need to be addressed to see if it is a good fit? Experts each received $50 merchandise cards in appreciation for their time. In accordance with the CE studio model, meals also were provided in the form of $30 meal cards (due to meeting virtually).

The CELS took brief notes throughout each CE studio, listening for common key points shared by the experts. Once all studios had been conducted, the CELS reviewed these notes in order to identify overarching similar key points that could be synthesized in a single overall studio summary. Following the studio model and IRB requirements, we did not transcribe or formally analyze studio conversations. To ensure the most important points were correctly captured and organized, the CELS conducted member checking with studio facilitators and four experts.

### Outcomes

The total number of experts was 31, averaging 8 experts per CE studio and drawn from all 12 New Mexico Health Councils that were invited to participate.

Three key points were identified across all four CE studios specific to community needs for research participation. These were (in no particular order): 1) things that help with community research participation, 2) things that get in the way of community research participation, and 3) things researchers should consider when approaching New Mexican communities. Table 1 summarizes responses to these aggregate key points.

**Table 1.** Community engagement studio key-points summary and recommendations by experts

Things that help with community research participation:
When assessing whether a project is a good fit, evaluate the community’s interest and capacity to assist and/or be involved in the research. Provide training and capacity-building for research Be transparent about research funding and intentions Build familiarity and trust with communities Collaborate with community champions and influencers Recruit through trusted community members and health councils Be available and responsive to community needs Bring the results of the study back to the community
Things that get in the way of community research participation:
Geographical barriers Internet access limitations Capacity issues Language barriers Time off work for participation Restrictions by grant funder Misinformation and cynicism in community about research Mental health-related barriers to participation (e.g., anxiety) Negative perceptions of research in certain communities Trust issues related to politics, the government, and certain topics
Things researchers should consider when approaching New Mexican communities:
The uniqueness of rural communities Cultural aspects of various populations in New Mexico such as Hispanic, Indigenous, Black, Veteran, Elder/Older, and Conservative populations Varying caregiver relationships such as relatives like aunts, grandparents, siblings, and foster parents

Regarding the first key point, *things that help with community research participation*, experts shared a variety of techniques and strategies that researchers could employ. They suggested that *when assessing whether a project is a good fit, evaluate the community’s interest and capacity to assist and/or be involved in the research*. Next, they suggested *providing training and capacity building for research* and explained that not all community members have research education.

Experts also shared that researchers needed to be *transparent about research funding and intentions.* These factors set the agenda for the research that is conducted and are important in determining individual and community research participation. In addition, experts suggested that researchers also need to *build familiarity and trust with communities*. In this context, experts from the Northwest region explained the need for “kinship” between researchers and community members. While kinship is a word often used to explain familial relationships, experts encouraged a similar type of relationship building for researchers to facilitate trust and break down historical barriers and challenges to research participation. Experts additionally stressed the need for researchers to *collaborate with community champions and influencers*, *recruit through trusted community members and health councils*, and *be available and responsive to community needs*. One expert explained how unresponsiveness to questions they had about a prior research survey they were being asked to complete led them to ultimately decide to not participate in that study. The important practice of *bringing the results of the study back to the community* was also strongly encouraged as a way for individuals to see the impact of their participation and also its benefit to the community.

The second key point that experts shared was *things that get in the way of research participation*. Geographical barriers, internet access limitations, and language barriers are challenges for research participation. Experts also shared that *capacity issues* in communities stood in the way of research participation as there are often not enough clinics, staff, or even public spaces to conduct research in their communities. In addition, experts shared that their communities are over-surveyed, so researchers should ensure needed data has not already been collected to optimize capacity. *Time off from work* also stood in the way of research participation. *Restrictions by the grant funders* were noted as a common challenge, especially when it comes to incentives for research participation such as providing a meal or cash outside of merchandise cards for participants. *Misinformation and cynicism about research* were lingering issues resulting from the COVID-19 pandemic the experts saw in their communities, followed by negative perception of research. *Trust issues related to politics, the government, and certain politically charged topics* also got in the way of research participation in their communities.

The final key point experts shared was *things researchers should consider* to enhance community research participation. Experts suggested that researchers need to *consider the uniqueness of rural communities* as some explained they chose to live in their rural communities for many reasons. They also offered that *cultural aspects of various populations in New Mexico need to be taken into consideration.* Experts mentioned *Hispanic, Indigenous, Black, veterans, elder/older, and conservative populations*. There are also *varying caregiver relationships that should be acknowledged and considered such as relatives like aunts, grandparents, siblings, and foster parents* rather than making nuclear family assumptions. Experts suggested that *Mental health, medical care, food deserts, and housing issues could affect research participation* as well and researchers should find ways to mitigate these issues to ease the added load of research participation burden for communities.

## Discussion

The community engagement CE studios provided novel information related to facilitators, barriers, and considerations for enhancing community engagement in health research and clinical trials participation. The concept of fostering “kinship” between researchers and community members as a means to facilitate trust and overcome historical barriers to research participation is new, and to the best of our knowledge, not captured in the community engagement literature. We also acknowledge this concept is not new to indigenous communities and has been a part of life since time immemorial. Second, through the studios we were able to recognize the uniqueness of rural communities and critical cultural diversities among New Mexico populations, as well as to more clearly visualize the spectrum of caregiver relationships, and socioeconomic challenges in this region. Third, our studios allowed us to hold a space where communities share their own personal and communal perspectives regarding what they see as the needs of and gaps in research participation in New Mexico. This tailored approach acknowledges the diversity within communities and emphasized the importance of culturally responsive and contextually relevant research practices.

The key points of our studios align with established principles in community engagement literature, particularly the Community-based Participatory Research (CBPR) approach. CBPR emphasizes collaboration between researchers and community members throughout the research process [4]. Key CBPR principles, reflected in our studios, include promoting active community engagement (*establish “kinship”*), capacity building (*provide training in research*), cultural humility (*consider uniqueness of each community and the cultures within*), focusing on local relevance (*assess community fit*), dissemination and knowledge sharing (*bring results back*), sustainable relationships (*build familiarity and trust*), and ethical considerations (*be transparent regarding funding and intentions*) [4,5,17]. Despite these principles being standard for community engagement, their reinforcement by community members suggests a continued need for researchers to implement these practices. Although these strategies are recognized as best practices in the literature, their full impact on communities has yet to be realized.

A key point that was highlighted often by the experts was the need for researchers to bring the results of the study back to communities. Honoring community feedback, the CELS conducted five verbal presentations to community health councils about the key points from the studios. In addition, the team created a two-pager infographic and shared it via email with all experts who had participated in the studios. Also, because key points were directed toward researchers, the CELS gave a presentation to leadership at the UNM HSC to bring awareness that the studios had occurred and to provide a call to action to researchers to consider community’s expert voices on best community engagement practices. An important suggestion from UNM HSC leadership was to publish this work to provide a citation that could underscore the CE studios’ key points.

In conclusion, our community engagement studios provided valuable insights for enhancing community engagement in health research and clinical trials. Our findings highlight the importance of recognizing the uniqueness of rural communities, cultural considerations, and socioeconomic challenges within diverse populations in New Mexico. By aligning with established principles in community engagement literature, particularly the CBPR framework, we have underscored the significance of promoting active community engagement, capacity building, cultural humility, and ethical considerations in research endeavors. Despite the recognition of these principles as best practices, our findings emphasize the ongoing need for researchers to implement and reinforce these practices to fully realize their impact on communities. Moving forward, it is imperative that we continue to prioritize community-driven approaches and collaborative partnerships to ensure equitable and meaningful participation in health research and clinical trials across diverse communities.
